# Supplementary figures and images for: The community acceptance of COVID-19 vaccines in Rakhine State: A cross-sectional study in Myanmar
Source: PLOS Glob Public Health. 2023 Aug 3;3(8):e0002162. doi: 10.1371/journal.pgph.0002162 (PMC10399871; doi:10.1371/journal.pgph.0002162)

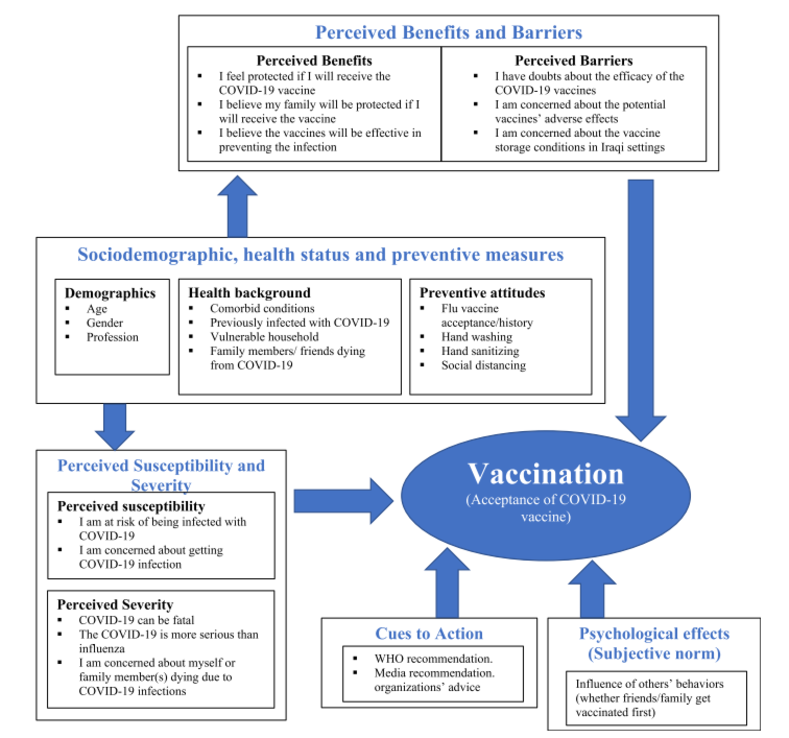

Supplement: S1 Fig — (TIF) [file pgph.0002162.s001.tif]
