# Supplementary material for: The community acceptance of COVID-19 vaccines in Rakhine State: A cross-sectional study in Myanmar
Source: PLOS Glob Public Health. 2023 Aug 3;3(8):e0002162. doi: 10.1371/journal.pgph.0002162 (PMC10399871; doi:10.1371/journal.pgph.0002162)
Supplement: Attachment — Submitted filename: Response to Reviewers.docx [file pgph.0002162.s002.docx]

Review Comments to the Author

**Reviewer #1: Thanks for the invitation to review this manuscript.
In the current study, the authors investigated the issue of COVID-19 vaccine acceptance and its associated determinants in Rakhine State, Myanmar.
The importance of this study can be related to the previous scarcity of such reports from Myanmar as highlighted previously in the recent review: https://doi.org/10.2147/JMDH.S347669, cited by the authors.
Therefore, the current study present original data on a timely and important public health issue despite the decline in interest in the research involving COVID-19 vaccine hesitancy.
The acceptance rate of COVID-19 vaccination in the study was high, which was in line of the results from countries in South East Asia.
Overall, the manuscript is well written with comprehensive literature review. The methodology is valid and the results were presented clearly and supported the conclusions.
Importantly, the authors elaborated well on the potential limitations of the study.
Therefore, I think that the manuscript can be considered for publication.
Congratulations!**

Author: Thank you for your kind comments. Your review and comments encourage the researchers in Myanmar to work on and prepare more research manuscripts in the future.

**Reviewer #2: Introduction/Background: Consider deleting or editing down lines 73-98 and 108-179. While these sections are well-written and informative, the paper would benefit from a tighter focus on Myanmar itself. Consider replacing this language with additional details on Myanmar's public health infrastructure, vaccination challenges, etc.**
Author: Edited and trimmed down lines 73-98 and also on line 108-179. The focus on Myanmar was described on the session “*Current Situation of COVID-19, Prevalence of Protective Behaviour and Progress of Vaccination in Myanmar*” with adding public health services and political situation effecting the vaccination program.

**Line 190: Figure 1 appears to be missing from the manuscript.**

Author: Already submitted as separate attachment. Now added to the manuscript. *Figure 1,2 3 and supplementary 1 (HBM elements) were added as separate attachments.*

**Re: additional figures: Consider including a map of Myanmar that highlights where the study was conducted.**

Author: Added the map of Rakhine State and selected study townships

**In the methods section, please explain what the health belief model is, and why it was chosen to analyze the findings from this study.**

*Author: Added explanation on the health belief model and the reason for choosing this model*

**Participants (Line 284): Please define "household leader" - does this refer to a male head of household?**

Author: Defined the household leader in the manuscript. “The household leader is the individual who serves as the main breadwinner or primary source of income in a household. This person is typically identified as the male husband in Myanmar, although in cases where the husband has passed away, the role may be filled by a female housewife.”

**Discussion: Please elaborate on how the findings of this study could inform vaccine delivery, risk communication, and/or community engagement around vaccination in Myanmar. In other words, what implications does this study have for public health policymaking and practice?**

Author: Implications of the study with possible implementations to increase the vaccine acceptance and improve the vaccination services in Myanmar were added in line 586 – 614 *(in revised manuscript without track changes)*.

**I would recommend moving the limitations to the discussion section instead of listing them after the conclusion. Additionally, consider commenting on the health belief model itself -- do you believe it is a sufficient tool for studying vaccine acceptance?**

Author: Moved the limitation to under the discussion session.

Yes, health belief model is a comprehensive and appropriate tool for studying vaccine acceptance. The hypothesis was tested by Karl et al. regarding relevance and effectiveness of HBM on COVID-19 predicting preventive behavior during COVID-19 pandemic (1). Multiple published studies, that are exploring the vaccine acceptance and hesitancy of population, are adapting the HBM. The elements of HBM and their influence on COVID-19 vaccination of an individual were illustrated by Al-Metawali et al in the figure below (2) (also added to supplementary material of manuscript with proper referencing).

*Figure: Health belief model elements and variables which have the potential to influence people's acceptance of receiving a COVID-19 vaccine*

Source: Al-Metwali et al. (2021)

References

1.	Karl JA, Fischer R, Druică E, Musso F, Stan A. Testing the Effectiveness of the Health Belief Model in Predicting Preventive Behavior During the COVID-19 Pandemic: The Case of Romania and Italy. Front Psychol [Internet]. 2022 Jan 12 [cited 2023 Mar 9];12:6454. Available from: https://www.frontiersin.org/articles/10.3389/fpsyg.2021.627575/full

2.	Al-Metwali BZ, Al-Jumaili AA, Al-Alag ZA, Sorofman B. Exploring the acceptance of COVID-19 vaccine among healthcare workers and general population using health belief model. J Eval Clin Pract [Internet]. 2021 Oct 1 [cited 2021 Nov 2];27(5):1112–22. Available from: https://onlinelibrary.wiley.com/doi/full/10.1111/jep.13581

***Author: The figure files were uploaded to the Preflight Analysis and Conversion Engine (PACE) digital diagnostic tool. And downloaded and attached in the revised manuscripts to make publication ready figures.***
