# Supplementary material for: The community acceptance of COVID-19 vaccines in Rakhine State: A cross-sectional study in Myanmar
Source: PLOS Glob Public Health. 2023 Aug 3;3(8):e0002162. doi: 10.1371/journal.pgph.0002162 (PMC10399871; doi:10.1371/journal.pgph.0002162)
Supplement: Attachment — Submitted filename: Response to Reviewers.docx [file pgph.0002162.s003.docx]

**Reviewer #1:** The manuscript provides an accurate and detailed analysis of the research conducted. Therefore, I endorse the manuscript for publication. Best wishes!

**Author:** Thank you for the comments and endorsement for publication.

**Reviewer #3:** The study presents primary and original data from the region, and the methodology, despite having some limitations, allows the authors to achieve their proposed objectives. However, the text contains many words, tables, and figures, making it time-consuming to read. Therefore, the authors should consider removing some of the unnecessary explanations from the introduction and methodology sections, which would help the reader focus on the main content and make the reading more fluid. Additionally, the limitations should be restated since they have an impact on the results.

Below are some suggestions and questions for the authors:

Line 41 - It would be helpful to convert the monthly income to US dollars to enable readers from other parts of the world to understand.
**Author:** Added the USD amount

Lines 90 to 159 - The information presented in this section doesn't add solidity to the manuscript. If the authors consider it important, they could present it as supplementary material. Alternatively, some of the information presented could be used in the introduction to make the reading more fluid and focused on the objective of the work.

**Author:** Summarize the key points from line 90 to 159. Only “Introduction” section was mentioned.

Research Design - The explanation of the Health Belief Model (HBM) seems unnecessary. The authors can cite references on the subject or consider it as supplementary material. This section should be objective, with the authors only stating what type of design was used in the study. The text from line 185 must be included in the results section.

**Author:** The explanation of HMB was mentioned due to the comments of reviewer 2 in prevision revision. Now removed the explanation.

Line 219 - It's unclear what "N4Studies" means.

**Author:** N4Studies is a mobile application for calculating sample size in research studies. (<https://play.google.com/store/apps/details?id=com.chetta.n4Studies&hl=en&gl=US&pli=1>)

Sampling Procedure - The text contains many justifications and explanations. The authors should only say **how the sampling process was carried out**, as between lines 237-240. This is one of the main limiting aspects of the study, which should be highlighted in the section "Limitations of Study" (line 738), which has been deleted.

**Author:** Only key procedure was mentioned.

Participants - The authors should rename this section to "Eligibility Criteria" and clearly state who was included in the study (and the criteria followed) and who was excluded (and the criteria followed).

**Author:** Renamed the section

Data Collection Tool - Were the questionnaires printed or completed on a mobile device? If printed, how were the data digitized after answering the questions? Was any quality control procedure adopted for this typing? Where were the completed questionnaires stored?

**Author:** The questionnaires were printed out and asked to the participants by the interviewer.

Data Entry and Analysis - Were the questionnaires printed or completed on a mobile device? If printed, how were the data digitized after answering the questions? Was any quality control procedure adopted for this typing? Where were the completed questionnaires stored?
**Author:** The questionnaires were printed out and asked to the participants by the interviewer. The data entry was done by Microsoft Excel. Cross-checking of electronic data against paper-based documents was performed for the data quality control. (Mentioned in the Data collection tool and Data entry and analysis section.)

Ethical Considerations - The authors should limit themselves to the ethical approvals obtained. The text between lines 273-278 should be deleted. If essential for methodological understanding, explanations of how the interviews were carried out (lines 284-289) should not appear in this section. If ethical approval opinions have any identifiers such as a numeric or alphanumeric code, they should be presented.

**Author:** Removed the lines 273-278 and 284-289. Numeric code of ethical approval was presented.


Line 305 - It's unclear why 276 participants were included when the sample calculation (line 221) predicted 288.

**Author:** Out of the targeted 288 participants, 276 were successfully interviewed. Unfortunately, rural households from Toungup township could not be assessed.

Line 349 - The authors need to add the unit of measurement ("108,000 and 200,000 per month").

**Author:** Added

Table 1 - The "Income" unit of measurement could be in US dollars to enable readers from other regions of the world to understand better. In the "Health Related Sector" variable, only the "No" answer should be presented.

**Author:** Added the USD amount. Deleted the answer “Yes” row.

Lines 329/330 - The text is repetitive and has already been mentioned in the "Data Entry and Analysis" section. Therefore, it should be removed.
**Author:** Removed the text

Influencing Factors for the Acceptance of COVID-19 Vaccines - The authors should incorporate these data into the section "Factors Associated with Acceptance of COVID-19 Vaccines."

**Author:** The two titles are different in analysis performed. The associated factors were calculated with Chi square while influencing factors were calculated with logistic regression analysis. So, the results should be described in different table.

In the original version the authors present a limitations section (lines 738-747), which does not appear in the current version. The study presents original and relevant data for the region, but it has limitations that should be highlighted, so that the reader can contextualize the findings.

**Author:** Limitation section was moved into discussed section due to the comment of reviewer 2 in previous revision. Now, the limitation section was presented.
